# Supplementary material for: Differential protein expression profiles in human sperm from teratozoospermic and normozoospermic men identify LTBP1 and TGF-βR1 as potential biomarkers within the TGF-β signalling pathway
Source: PLoS One. 2026 Feb 13;21(2):e0342465. doi: 10.1371/journal.pone.0342465 (PMC12904376; doi:10.1371/journal.pone.0342465)
Supplement: S1 Table — (DOCX) [file pone.0342465.s001.docx]

**S1 Table. The list of the 39 overlapping proteins in the TGF-β signalling pathway in human sperm (TER versus NOR)**

| **Accession number** | **Protein name** | **Gene symbol** | **Log2 fold change^a^ (TER/NOR)** | **p-value** | **q-value** |
| --- | --- | --- | --- | --- | --- |
| Q9HCE7 | E3 ubiquitin-protein ligase SMURF1 | SMURF1 | -2.529 | 0.165 | 0.711 |
| Q6ZVN8 | Hemojuvelin | HJV | -1.075 | 0.059 | 0.570 |
| Q13145 | BMP and activin membrane-bound inhibitor homolog | BAMBI | -0.882 | 0.145 | 0.693 |
| P30154 | Serine/threonine-protein phosphatase 2A 65 kDa regulatory subunit A beta isoform | PPP2R1B | -0.765 | N/A | N/A |
| O95405 | Zinc finger FYVE domain-containing protein 9 | ZFYVE9 | -0.662 | 0.672 | 0.890 |
| Q13464 | Rho-associated protein kinase 1 | ROCK1 | -0.458 | 0.545 | 0.847 |
| O15198 | Mothers against decapentaplegic homolog 9 | SMAD9 | -0.447 | 0.611 | 0.870 |
| P08476 | Inhibin beta A chain | INHBA | -0.229 | 0.635 | 0.878 |
| P23443 | Ribosomal protein S6 kinase beta-1 | RPS6KB1 | -0.223 | 0.764 | 0.919 |
| P36896 | Activin receptor type-1B | ACVR1B | -0.156 | 0.801 | 0.930 |
| P19883 | Follistatin | FST | -0.106 | 0.863 | 0.953 |
| Q9HAU4 | E3 ubiquitin-protein ligase SMURF2 | SMURF2 | -0.092 | 0.707 | 0.900 |
| Q09472 | Histone acetyltransferase p300 | EP300 | -0.070 | 0.772 | 0.922 |
| P03971 | Muellerian-inhibiting factor | AMH | -0.019 | 0.953 | 0.988 |
| Q8N6C5 | Immunoglobulin superfamily member 1 | IGSF1 | 0.117 | 0.704 | 0.898 |
| Q9H2X0 | Chordin | CHRD | 0.148 | 0.787 | 0.925 |
| P27037 | Activin receptor type-2A | ACVR2A | 0.156 | 0.718 | 0.903 |
| Q6ZMP0 | Thrombospondin type-1 domain-containing protein 4 | THSD4 | 0.170 | 0.620 | 0.872 |
| Q7Z4P5 | Growth/differentiation factor 7 | GDF7 | 0.224 | 0.591 | 0.864 |
| P42772 | Cyclin-dependent kinase 4 inhibitor B | CDKN2B | 0.251 | 0.207 | 0.744 |
| P81172 | Hepcidin | HAMP | 0.270 | 0.301 | 0.784 |
| Q13873 | Bone morphogenetic protein receptor type-2 | BMPR2 | 0.341 | 0.250 | 0.765 |
| P07996 | Thrombospondin-1 | THBS1 | 0.355 | 0.285 | 0.778 |
| Q92859 | Neogenin | NEO1 | 0.471 | N/A |  |
| P36894 | Bone morphogenetic protein receptor type-1A | BMPR1A | 0.528 | 0.244 | 0.762 |
| O00292 | Left-right determination factor 2 | LEFTY2 | 0.533 | 0.321 | 0.787 |
| Q92793 | CREB-binding protein | CREBBP | 0.571 | 0.294 | 0.783 |
| O15105 | Mothers against decapentaplegic homolog 7 | SMAD7 | 0.571 | 0.227 | 0.751 |
| P27361 | Mitogen-activated protein kinase 3 | MAPK3 | 0.635 | 0.421 | 0.816 |
| Q04771 | Activin receptor type-1 | ACVR1 | 0.695 | 0.114 | 0.655 |
| Q6NW40 | Repulsive guidance molecule B | RGMB | 0.717 | 0.071 | 0.600 |
| Q8NER5 | Activin receptor type-1C | ACVR1C | 0.813 | 0.170 | 0.716 |
| P58166 | Inhibin beta E chain | INHBE | 0.825 | 0.390 | 0.810 |
| P61812 | Transforming growth factor beta-2 proprotein | TGFB2 | 0.905 | 0.055 | 0.559 |
| P10600 | Transforming growth factor beta-3 proprotein | TGFB3 | 0.924 | 0.068 | 0.597 |
| Q13705 | Activin receptor type-2B | ACVR2B | 1.179 | 0.216 | 0.749 |
| P35555 | Fibrillin-1 | FBN1 | 1.205 | 0.150 | 0.696 |
| O43541 | Mothers against decapentaplegic homolog 6 | SMAD6 | 1.412 | 0.508 | 0.843 |
| Q14766 | Latent-transforming growth factor beta-binding protein 1 | LTBP1 | 1.681 | 0.020 | 0.433 |

## ^a^ Values greater than 0 indicate up-regulation, while values less than 0 indicate down-regulation.
