# Supplementary material for: Differential protein expression profiles in human sperm from teratozoospermic and normozoospermic men identify LTBP1 and TGF-βR1 as potential biomarkers within the TGF-β signalling pathway
Source: PLoS One. 2026 Feb 13;21(2):e0342465. doi: 10.1371/journal.pone.0342465 (PMC12904376; doi:10.1371/journal.pone.0342465)
Supplement: Attachment — Submitted filename: Review Report- PLOS ONE .docx [file pone.0342465.s002.docx]

**Review Report**

**Title:** Differential protein expression profiles in human sperm from teratozoospermic and normozoospermic men: A spotlight on proteins in the TGF-β signalling pathway

**Strengths**

1. **Relevant topic:** Male infertility and sperm proteomics are of significant clinical and scientific interest.
2. **Novelty:** Focus on TGF-β pathway proteins (LTBP1 and TGF-βR1) in teratozoospermia is interesting and could provide biomarker insights.
3. **Methodology:** Use of LC-MS/MS and immunocytochemistry adds robustness through complementary approaches.
4. **Ethics & data:** Proper IRB approval, consent, and data availability are reported.

**Major Issues (Require Revision)**

1. **Sample size and statistical power:**
   - Only 42 men included (20 NOR, 22 TER). This is underpowered for proteomic studies reporting 9,000+ proteins. Authors should justify sample size and power.
   - Number of samples used in proteomics vs immunocytochemistry is inconsistent (not all 42 analyzed). Needs clarification.
2. **Data presentation and filtering:**
   - Reported *9,088 DEPs* between groups seems biologically unrealistic given small n. Criteria for defining DEPs (fold change, p-value/FDR) are unclear.
   - Cutoff of “1.0 fold change” is misleading (does this mean log2 fold = 1? or just ≥1.0 difference?). Needs proper explanation.
   - No multiple testing correction (e.g., Benjamini–Hochberg) applied in proteomic analysis → high risk of false positives.
3. **Validation of findings:**
   - Only two proteins (LTBP1, TGF-βR1) are discussed/validated, while 39 DEPs in TGF-β pathway were identified. Why were others ignored?
   - Western blot or quantitative validation (qPCR, targeted proteomics) is missing. Immunocytochemistry alone is insufficient for robust validation.
4. **Interpretation / causality:**
   - Discussion often overstates causality (e.g., suggesting therapeutic targets) based only on correlation data. Needs more cautious language.
   - Biological plausibility of linking LTBP1/TGF-βR1 directly to teratozoospermia is not fully developed. Other confounders (age, lifestyle, comorbidities) not addressed.
5. **Figures / tables:**
   - Figures are difficult to interpret (low resolution, not well labeled).
   - S1 Table with 39 DEPs should include statistical significance (p/FDR values), fold change, accession IDs.
6. **Language and clarity:**
   - Manuscript has frequent grammatical issues and redundant statements. Needs thorough English editing for clarity and conciseness.

**Minor Issues**

1. Abstract mentions *“cut-off 1.0 fold change”* → should be clarified (likely log2FC).
2. Methods: Proteomic workflow details are too technical for PLOS ONE (e.g., buffer recipes). Focus should be on reproducibility and data processing.
3. Data availability statement should specify raw proteomics files deposited in a public repository (e.g., PRIDE), not only “within manuscript.”
4. References: Some older references (1990s, early 2000s) dominate. Update with recent proteomics literature (esp. 2022–2024).
5. Ethical statement: Consent type (written/oral) not clearly stated, as required by PLOS ONE.

**Recommendation**

**Decision: Major Revision (not reject).**

- The study has potential but currently lacks sufficient statistical rigor, validation, and clarity for publication.
- Authors must:
  - Clarify statistical thresholds and apply multiple-testing correction.
  - Justify sample size.
  - Provide stronger validation beyond immunocytochemistry.
  - Improve figure/table presentation.
  - Revise language for clarity and avoid overinterpretation.

If these issues are addressed, the manuscript could be suitable for publication in PLOS ONE, which accepts technically sound work even if incremental. In its current form, it is not acceptable without substantial revision.
