# Supplementary material for: Differential protein expression profiles in human sperm from teratozoospermic and normozoospermic men identify LTBP1 and TGF-βR1 as potential biomarkers within the TGF-β signalling pathway
Source: PLoS One. 2026 Feb 13;21(2):e0342465. doi: 10.1371/journal.pone.0342465 (PMC12904376; doi:10.1371/journal.pone.0342465)
Supplement: Attachment — Submitted filename: Response to Reviewers_.docx [file pone.0342465.s004.docx]

**5. Review Comments to the Author**

**Reviewer #1:**

1. The title is specific, indicating both the biological system (human sperm), the condition studied (teratozoospermia), and the signaling pathway of interest (TGF-β). Consider specifying that LTBP1 and TGF-βR1 are identified as potential biomarkers to make the title more impactful.

**Author response:** In accordance with this recommendation, we have revised the title to specify that LTBP1 and TGF-βR1 are identified as potential biomarkers, thereby making the title more specific and impactful. The revised title now reads as follows: “Differential protein expression profiles in human sperm from teratozoospermic and normozoospermic men identify LTBP1 and TGF-βR1 as potential biomarkers within the TGF-β signalling pathway” [Lines 2-3].

2. The short title could be clearer: consider “TGF-β pathway proteins in teratozoospermic sperm” for better focus.

**Author response:** We appreciate the reviewer’s helpful suggestion. We have revised the short title to “TGF-β signalling pathway proteins in teratozoospermic sperm” to improve clarity and focus.

3. In Abstract: Highlight the novelty of the finding (i.e., LTBP1 and TGF-βR1’s correlation with sperm morphology) more explicitly.

**Author response:** We have revised the Abstract section to more explicitly highlight the novelty of our findings, emphasizing the correlation of LTBP1 and TGF-βR1 with sperm morphology, as stated: “Our findings, for the first time, demonstrate a significant association between the expression of LTBP1 and TGF-βR1 and abnormal sperm morphology, supporting their consideration as novel exploratory biomarkers for further investigation in infertility, especially in TER men.” [Lines 37-40].

4. The introduction could more directly state the hypothesis or research question. Consider summarizing the literature review more concisely to keep the narrative focused.

**Author response:** We have revised the Introduction section to more clearly state the hypothesis and research question, as stated: “Even though extensive research has been done on sperm protein biomarkers, understanding of the proteomic alterations underlying male infertility remains limited, particularly in cases of TER. The potential involvement of the TGF-β signalling pathway in regulating sperm morphology has been hypothesized but not yet clearly elucidated.” [Lines 79-82]. Additionally, we have condensed the literature review to focus on the most relevant studies, improving the clarity and flow of the narrative.

5. Provide the objective as a standalone sentence, possibly in a separate subheading to enhance clarity for readers and reviewers.

**Author response:** We thank the reviewer for highlighting this important point. In response, we have revised the manuscript to present the study objective as a standalone sentence under a separate subheading, thereby improving clarity for readers and reviewers, as stated: “Therefore, this study aimed to investigate the proteomic profile of human sperm from TER men, with a focus on identifying key proteins within the TGF-β signalling pathway that may contribute to abnormal sperm morphology.” [Lines 83-85].

6. A methodology flowchart or summary table would improve readability. Expand slightly on how missing data were handled (e.g., why a constant zero was chosen for imputation).

**Author response:** In the Methods section, we have clarified that missing values in the proteomics analysis were handled using Perseus software (version 1.6.6.0) with a constant value of zero, as stated: “Missing values were handled using Perseus software (version 1.6.6.0) by imputing a constant zero for proteins not detected in all samples, providing a consistent baseline for statistical analysis [22].” [Lines 128-130].

7. Statistical analysis: Clarify the number of biological replicates used for all analyses. Apply or justify the absence of multiple comparison correction for the proteomics dataset.

**Author response:** In this study, sperm samples from 20 NOR and 22 TER men were analysed as biological replicates for proteomic analysis, as stated: “The human sperm protein was extracted from each semen sample (n = 20 NOR and n = 22 TER).” [Lines 108-109]. Results from proteomics analysis identified LTBP1 as a key protein involved in the TGF-β signalling pathway under TER conditions. Among the analysed samples, LTBP1 expression was detected in 14 samples from the NOR group and 16 samples from the TER group. Therefore, these samples were selected for subsequent protein validation using immunocytochemistry to investigate the relationship between LTBP1 and TGF-βR1. This information has been clearly stated in the Methods section as follows: “Sperm samples from 14 NOR and 16 TER men exhibiting detectable LTBP1 expression were selected for validation analysis using immunocytochemistry” [Lines 143-144].

Moreover, we used a relatively small sample size and an exploratory proteomic approach aimed at identifying potential candidate proteins for further validation. Therefore, we presented unadjusted p-values while clearly acknowledging the potential for false positives. This limitation has now been stated clearly in the revised manuscript (Discussion section). The Discussion section has been updated, as stated: “Statistical analyses were performed without multiple testing correction due to the exploratory purpose and limited sample size of the dataset.” [Lines 264-266].

8. Consider reordering figures to follow the sequence of presentation in the results. A summary table of key proteins with function annotations would enhance accessibility.

**Author response:** We have carefully checked the figures and confirmed that their order appropriately follows the sequence of presentation in the Results section. Regarding the summary table, since this study focuses on KEGG pathways as the primary functional annotations, we did not include additional annotations such as Biological Process, Molecular Function, or Cellular Component for each protein. However, we have provided the Accession numbers in the table, which allow readers to access these additional functional annotations if desired.

9. Strengthen the discussion of limitations: e.g., cross-sectional nature, lack of functional assays. Briefly propose next steps (e.g., validating biomarkers in larger cohorts or mechanistic studies).

**Author response:** The limitations of the study have now been clearly stated in the revised manuscript (Discussion section), including the absence of multiple testing correction in the statistical analyses and the need for further validation. In addition, we have proposed future directions, including validating the identified biomarkers in larger independent cohorts and performing mechanistic studies to further elucidate their roles in sperm morphology regulation, as stated: “Validation of LTBP1 and TGF-βR1 as biomarkers in larger populations and mechanistic studies exploring their roles affecting sperm morphology will strengthen the translational potential of these findings.” [Lines 325-327].

10. In conclusion: Reinforce how this work fills the previously identified gap

**Author response:** We thank the reviewer for providing this valuable guidance. We have revised the Conclusion section to more explicitly highlight how our study addresses the previously identified gap, as stated: “We suggest that LTBP1 and TGF-βR1 proteins have the most impact as sperm protein biomarkers defining infertility related to abnormal sperm morphology. Notably, our findings provide novel evidence supporting the investigation of proteomic profiles within the TGF-β signalling pathway, highlighting their potential relevance as sperm biomarkers for infertility, particularly in the case of TER.” [Lines 348-352].

**Reviewer #2**

**Major Issues (Require Revision)**

**1. Sample size and statistical power:**

- Only 42 men included (20 NOR, 22 TER). This is underpowered for proteomic studies reporting 9,000+ proteins. Authors should justify sample size and power.

**Author response:** We acknowledge that the sample size in the present study is relatively limited for large-scale proteomic analysis. However, the sample size was determined based on prior power calculations primarily optimized for gene expression analysis using immunohistochemistry, which served as the main validation approach in this study. To clarify our sample-size rationale, we performed standard power calculations for an independent two-sample, two-tailed t-test (α = 0.05). The required sample size is highly dependent on the expected effect size (Cohen’s d). Based on standard benchmarks, detecting a large effect (d = 0.8) at a statistical power between 0.6 and 0.8 requires approximately 16-25 participants per group. Therefore, our sample size (20 NOR, 22 TER) provides adequate power to detect large effect-size changes. Notably, our study followed a discovery-to-validation workflow. After the proteomic discovery phase, we targeted molecular validation on the best candidates. We used strict quality control and filtering to focus on proteins that had significant changes that were biologically meaningful.

Our study design is consistent with several prior human proteomic investigations that successfully utilized modest sample sizes (typically 8–10 samples per group) during the discovery phase and still identified biologically relevant and statistically robust differential proteins, particularly when complemented by downstream validation. For example, Lee et al. (2024) demonstrated through simulation analyses that small group sizes (n = 8-13) are commonly applied in discovery or proof-of-concept studies, acknowledging that while statistical power for small effect sizes may be limited, such designs remain effective for identifying large and biologically relevant differences [1]. Similarly, Wang et al. (2019) analysed plasma proteomes from eight young and aged individuals and detected significantly differentially expressed proteins with adequate statistical control [2]. Furthermore, Liu et al. (2012) reported that a group size of 10 (male vs. female) achieved approximately 95% completeness of qualitatively identified urinary proteins and peptides [3]. Collectively, these studies support the appropriateness of our sample size for preliminary proteomic discovery.

References

1. Lee KH, Assassi S, Mohan C, Pedroza C. Addressing statistical challenges in the analysis of proteomics data with extremely small sample size: a simulation study. BMC Genomics. 2024;25(1):1086. doi: 10.1186/s12864-024-11018-2.
2. Wang H, Zhu X, Shen J, Zhao E-F, He D, Shen H, et al. Quantitative iTRAQ-based proteomic analysis of differentially expressed proteins in aging in human and monkey. BMC Genomics. 2019;20(1):725. doi: 10.1186/s12864-019-6089-z.
3. Liu X, Shao C, Wei L, Duan J, Wu S, Li X, et al. An individual urinary proteome analysis in normal human beings to define the minimal sample number to represent the normal urinary proteome. Proteome Science. 2012;10(1):70. doi: 10.1186/1477-5956-10-70.

- Number of samples used in proteomics vs immunocytochemistry is inconsistent (not all 42 analysed). Needs clarification.

**Author response:** Results from proteomics analysis identified LTBP1 as a key protein involved in the TGF-β signalling pathway under TER conditions. Among the samples analysed, LTBP1 expression was detected in 14 samples from the NOR group and 16 samples from the TER group. Therefore, only these samples were selected for subsequent protein validation using immunohistochemistry to investigate the relationship between LTBP1 and TGF-βR1.

**2. Data presentation and filtering:**

- Reported *9,088 DEPs* between groups seems biologically unrealistic given small n. Criteria for defining DEPs (fold change, p-value/FDR) are unclear.

**Author response:** Originally, the *9,088 DEPs* referred to proteins that were detected in both the NOR and TER groups, regardless of whether they were differentially expressed. However, we recognize that using **DEPs** for this explanation was inaccurate. Therefore, we have revised it to **overlapping proteins** for clarity and a more accurate description.

- Cutoff of “1.0 fold change” is misleading (does this mean log2 fold = 1? or just ≥1.0 difference?). Needs proper explanation.

**Author response:** We thank the reviewer for this helpful suggestion. We have revised the text to clarify that differences in protein expression levels between experimental conditions are quantified using **the log2 fold change.** This clarification has been incorporated into the revised manuscript including:

- The Abstract section has been updated as follows: “Six proteins that were differentially expressed and had a log2 fold change of ≥ 1 or ≤ -1 were considered to be the differentially expressed proteins in human sperm between the groups.” [Lines 30-32].
- The Results section has been updated as follows: “Six of the 39 overlapping proteins with a log2 fold change ≥ 1 or ≤ -1 were defined as DEPs.” [Lines 183].
- Both Table 1 and S1 Table have been revised [Lines 191-193].
- No multiple testing correction (e.g., Benjamini–Hochberg) applied in proteomic analysis → high risk of false positives.

**Author response:** In this study, we used a relatively small sample size and an exploratory proteomic approach aimed at identifying potential candidate proteins for further validation. Therefore, we presented unadjusted p-values while clearly acknowledging the potential for false positives. This limitation has now been stated clearly in the revised manuscript (Discussion section). The Discussion section has been updated, as stated: “Statistical analyses were performed without multiple testing correction due to the exploratory purpose and limited sample size of the dataset.” [Lines 264-266].

**3. Validation of findings:**

- Only two proteins (LTBP1, TGF-βR1) are discussed/validated, while 39 DEPs in TGF-β pathway were identified. Why were others ignored?

**Author response:** Although 39 overlapping proteins were identified as part of the TGF-β signalling pathway, we focused on LTBP1 because it showed the most significant differential expression (log2 fold change = 1.681 and p = 0.02) as shown in Table 1. In addition, TGF-βR1 was included for validation due to its close functional relationship with LTBP1 and its reported association with sperm morphology.

- Western blot or quantitative validation (qPCR, targeted proteomics) is missing. Immunocytochemistry alone is insufficient for robust validation.

**Author response:** We appreciate the reviewer’s valuable comment. We agree that adding quantitative validations such as Western blot or qPCR would further strengthen the findings. However, our study focused on exploratory proteomics combined with immunocytochemistry to validate both the expression level and localization of candidate proteins. Immunocytochemistry in this study was performed as a **quantitative validation approach**, comparable to Western blot and qPCR. While immunocytochemistry provides additional advantages in demonstrating **specific localization and expression patterns of target proteins within sperm,** which is information that cannot be obtained from Western blot or qPCR. Therefore, we consider immunocytochemistry to provide strong and sufficient quantitative validation for the scope of this study. Consistent with our approach, several previous studies have utilized immunohistochemistry to validate their proteomics results, confirming its reliability as a complementary validation method [1, 2].

References

1. Richter A, Fichtner A, Joost J, Brockmeyer P, Kauffmann P, Schliephake H, et al. Quantitative proteomics identifies biomarkers to distinguish pulmonary from head and neck squamous cell carcinomas by immunohistochemistry. J Pathol Clin Res. 2022;8(1):33-47. doi: 10.1002/cjp2.244. PubMed PMID: 34647699; PubMed Central PMCID: PMCPMC8682946.
2. Ichimata S, Hata Y, Yoshinaga T, Katoh N, Kametani F, Yazaki M, et al. Amyloid-Forming Corpora Amylacea and Spheroid-Type Amyloid Deposition: Comprehensive Analysis Using Immunohistochemistry, Proteomics, and a Literature Review. Int J Mol Sci. 2024;25(7). doi: 10.3390/ijms25074040. PubMed PMID: 38612850; PubMed Central PMCID: PMCPMC11012059.

**4. Interpretation / causality:**

- Discussion often overstates causality (e.g., suggesting therapeutic targets) based only on correlation data. Needs more cautious language.

**Author response:** We have carefully revised the Discussion section to use more cautious and descriptive language, avoiding overinterpretation of the results.

- Biological plausibility of linking LTBP1/TGF-βR1 directly to teratozoospermia is not fully developed. Other confounders (age, lifestyle, comorbidities) not addressed.

**Author response:** We appreciate this insightful comment. At present, we are unable to identify any studies demonstrating a direct link between LTBP1 or TGF-βR1 and teratozoospermia. To our knowledge, no reports have yet established such a direct mechanistic relationship. However, we agree that this is an important issue that requires further exploration. We also justified that common confounders, such as age, lifestyle, and comorbidities, have a limited influence on teratozoospermia, which is consistent with prior reports. Age has been shown to have a minimal direct impact on sperm DNA fragmentation, which is related to sperm quality, but not specifically on teratozoospermia [1]. Another study reported that age varied significantly within individuals over time, but its direct impact on sperm morphology was not highlighted [2]. Moreover, our findings underscore the importance of focusing on genetic and molecular factors rather than lifestyle changes. This approach aligns with current research emphasizing the genetic basis of male infertility, particularly in conditions like teratozoospermia, where environmental factors may play a lesser role [3]. Additionally, studies investigating comorbidities such as metabolic syndrome and obesity did not find a direct correlation with abnormal sperm morphology [4, 5]. This perspective is supported by recent studies highlighting the genetic factors contributing to teratozoospermia, which reinforce the need for a genetic diagnosis and targeted therapies in male infertility [6]. Thus, our study did not address other confounders.

References

1. Chua SC, Yovich SJ, Hinchliffe PM, Yovich JL. Male Clinical Parameters (Age, Stature, Weight, Body Mass Index, Smoking History, Alcohol Consumption) Bear Minimal Relationship to the Level of Sperm DNA Fragmentation. J Pers Med. 2023;13(5). doi: 10.3390/jpm13050759. PubMed PMID: 37240929; PubMed Central PMCID: PMCPMC10221623.
2. Lyons HE, Gyawali P, Mathews N, Castleton P, Mutuku SM, McPherson NO. The influence of lifestyle and biological factors on semen variability. J Assist Reprod Genet. 2024;41(4):1097-109. doi: 10.1007/s10815-024-03030-y. PubMed PMID: 38294621; PubMed Central PMCID: PMCPMC11052966.
3. Kyrgiafini MA, Giannoulis T, Chatziparasidou A, Christoforidis N, Mamuris Z. Whole-Genome Profile of Greek Patients with Teratozοοspermia: Identification of Candidate Variants and Genes. Genes (Basel). 2022;13(9). doi: 10.3390/genes13091606. PubMed PMID: 36140773; PubMed Central PMCID: PMCPMC9498395.
4. Ventimiglia E, Capogrosso P, Colicchia M, Boeri L, Serino A, Castagna G, et al. Metabolic syndrome in white European men presenting for primary couple's infertility: investigation of the clinical and reproductive burden. Andrology. 2016;4(5):944-51. doi: 10.1111/andr.12232. PubMed PMID: 27368157.
5. Osadchuk LV, Kleshchev MA, Osadchuk AV. Association of Overweight and Obesity with Semen Parameters and Metabolic and Hormonal Status in Young Men. Bulletin of Experimental Biology and Medicine. 2023;175(6):744-8. doi: 10.1007/s10517-023-05937-5.
6. Chang Y, Jiang X, Liu W, Zhang D, Yang S, Zhao D. Molecular genetic mechanisms of teratozoospermia. Zygote. 2023;31(2):101-10. doi: 10.1017/s0967199422000594. PubMed PMID: 36740361.

**5. Figures / tables:**

- Figures are difficult to interpret (low resolution, not well labeled).

**Author response:** All figures have been updated and submitted in high-resolution format (600 dpi) in accordance with PLOS ONE publication requirements. We have also ensured that all symbols, abbreviations, and colour codes are consistently applied and clearly defined in the figure legends. Should any figures still appear unclear, we would be glad to revise them further as needed.

- S1 Table with 39 DEPs should include statistical significance (p/FDR values), fold change, accession IDs.

**Author response:** The revised S1 Table and Table 1 now include **the statistical significance values (p-values), as well as the log2 fold change and accession number** for all proteins [Lines 191-193]. These additions improve the transparency and reproducibility of our results.

**6. Language and clarity:**

- Manuscript has frequent grammatical issues and redundant statements. Needs thorough English editing for clarity and conciseness.

**Author response:** The entire manuscript has been carefully revised to correct grammatical errors and eliminate redundant statements. We have also improved sentence structure and overall readability to enhance clarity and conciseness. A native speaker has performed a professional language edit to ensure the manuscript meets high English writing standards.

**Minor Issues**

1. Abstract mentions *“cut-off 1.0 fold change”* → should be clarified (likely log2FC).

**Author response:** In our analysis, the cutoff of “1.0 fold change” refers specifically to a log2 fold change. We have clarified this in the revised manuscript to avoid misunderstanding. The Abstract section has been updated as follows: “Six proteins that were differentially expressed and had a log2 fold change of ≥ 1 or ≤ -1 were considered to be the differentially expressed proteins in human sperm between the groups.” [Lines 30-32].

2. Methods: Proteomic workflow details are too technical for PLOS ONE (e.g., buffer recipes). Focus should be on reproducibility and data processing.

**Author response:** The proteomic workflow details have been revised as suggested.

3. Data availability statement should specify raw proteomics files deposited in a public repository (e.g., PRIDE), not only “within manuscript.”

**Author response:** While we agree that public data sharing is important, the raw proteomics data for this study are part of an ongoing project and are being retained for further analysis. Therefore, they have not been deposited in a public repository at this time. All processed data supporting the findings of this study are included within the manuscript and supplementary materials. The raw data can be made available from the corresponding author upon reasonable request.

4. References: Some older references (1990s, early 2000s) dominate. Update with recent proteomics literature (esp. 2022–2024).

**Author response:** We have carefully updated the references and incorporated recent proteomics literature throughout the manuscript to ensure the citations reflect the latest advances in the field. Updated references have been incorporated into the reference list as items 16, 17, 18, 27, 31, 34, 35, 36, 41, and 52.

5. Ethical statement: Consent type (written/oral) not clearly stated, as required by PLOS ONE.

**Author response:** We thank the reviewer for pointing this out. We confirm that written informed consent was obtained from all participants, and the ethical statement in the manuscript has been updated to clearly reflect this. The methods section has been updated as follows: “Written informed consent was provided by each volunteer.” [Lines 94].

**Response to Editor:**

While the study addresses an important issue in infertile patients, the sample size seems to be on the lower side. Further, the rationale for choosing only two proteins among the many differentially expressed proteins needs strong justification. Validation of the identified proteins by Western blotting is essential. Tone down the discussion and limit to the actual observations.

**Author response:** The issues raised have been thoroughly addressed in our **Author Response to Reviewer #2**, under **Comment 1** (Sample size and statistical power) and **Comment 3** (Validation of findings) sections. Moreover, we have revised the Discussion section to moderate the interpretation of our findings and focus more directly on the actual observations derived from the data.

**Response to Journal Requirements:**

1. Please ensure that your manuscript meets PLOS ONE's style requirements, including those for file naming. The PLOS ONE style templates can be found at https://journals.plos.org/plosone/s/file?id=wjVg/PLOSOne_formatting_sample_main_body.pdf and https://journals.plos.org/plosone/s/file?id=ba62/PLOSOne_formatting_sample_title_authors_affiliations.pdf

**Author response:** We have carefully revised the manuscript to ensure compliance with PLOS ONE’s style and formatting requirements, including file naming and manuscript structure, following the templates provided by the journal.

2. Please provide additional details regarding participant consent. In the ethics statement in the Methods and online submission information, please ensure that you have specified (1) whether consent was informed and (2) what type you obtained (for instance, written or verbal, and if verbal, how it was documented and witnessed). If your study included minors, state whether you obtained consent from parents or guardians. If the need for consent was waived by the ethics committee, please include this information.

**Author response:** The Ethics Statement has been updated to include further details regarding participant consent. Each volunteer provided written informed consent, as we specified in the Methods section [Lines 94].

3. If the reviewer comments include a recommendation to cite specific previously published works, please review and evaluate these publications to determine whether they are relevant and should be cited. There is no requirement to cite these works unless the editor has indicated otherwise.

**Author response:** No reviewer comments included recommendations to cite specific previously published works. Therefore, no additional citations were required.
