# Supplementary material for: Differential protein expression profiles in human sperm from teratozoospermic and normozoospermic men identify LTBP1 and TGF-βR1 as potential biomarkers within the TGF-β signalling pathway
Source: PLoS One. 2026 Feb 13;21(2):e0342465. doi: 10.1371/journal.pone.0342465 (PMC12904376; doi:10.1371/journal.pone.0342465)
Supplement: Attachment — Submitted filename: Response to Reviewers (2nd revision).docx [file pone.0342465.s006.docx]

**6. Review Comments to the Author**

**Reviewer #1:**

The authors have appropriately addressed all of my comments, and I recommend that this manuscript be accepted for publication.

**Author response:** We sincerely thank the reviewer for the time and effort in reviewing our manuscript. We appreciate the positive feedback and the helpful suggestions provided during the revision process, which have significantly improved the quality of our work.

**Reviewer #2**

The authors have addressed several editorial and clarity issues; however, key scientific and compliance concerns remain.

1. First, the raw proteomics data are not deposited in a public repository, which does not meet PLOS ONE’s mandatory data availability requirements; this must be corrected by depositing raw LC-MS/MS files (e.g., PRIDE accession) or by providing a journal approved controlled-access justification.

**Author response:** We thank the reviewer for highlighting this important point. We have added in section of **Data availability**, as stated: “The LC-MS/MS datasets generated during this study are available in the ProteomeXchange repository under the accession IDs JPST004285 and PXD072557 (via jPOST: https://repository.jpostdb.org/preview/7054576026955c0503b3d8, Access key: 9498). Raw MALDI-TOF MS and clinical data of this study are available from the corresponding author upon reasonable request.”.

2. Second, the proteomics statistical analysis requires re-evaluation: multiple-testing correction (FDR) should be applied and reported, or an explicit and technically sound justification must be provided along with evidence that major findings (e.g., LTBP1) remain robust under alternative imputation and significance thresholds.

**Author response:** We appreciate the reviewer’s focus on statistical rigor. In accordance with the reviewer's suggestion, the Benjamini-Hochberg procedure was applied to the proteomic dataset to control the false discovery rate (FDR) for multiple comparisons. The results were reported as FDR-adjusted p-values (q-values), with a significant threshold set at an FDR of 0.01. In the initial proteomic screen, LTBP1 was identified as a candidate of interest based on its high fold-change and nominal significance, though it did not maintain significance after FDR correction. To rigorously test this finding, we performed independent validation via the reverse transcription-quantitative polymerase chain reaction (RT-qPCR). This targeted analysis confirmed a significant increase in LTBP1 mRNA levels, validating the biological relevance of the protein.

The statistical analysis in **the Methods section** has been updated as follows: “Multiple testing correction was performed using the Benjamini-Hochberg procedure, and results were reported as FDR-adjusted p-values (q-values), with a significance threshold set at an FDR of 0.01.” [Lines 176-178].

We updated **the Results section** to include the following: “We found that among these proteins, the LTBP1 protein expression was significantly increased in the TER group compared with the NOR group, with a log2 fold change of 1.681 (p < 0.05) (see Table 1 and Fig 3). Despite not reaching statistical significance following FDR correction, the LTBP1 exhibited a high fold-change and clear biological relevance in the proteomic screen. To verify this candidate, we performed independent validation using RT-qPCR, which successfully confirmed its differential expression.” [Lines 205-210]. We have revised Table 1 and S1 Table to include both the p-values and q-values for all reported proteins.

3. Third, the validation strategy remains insufficient. Immunocytochemistry alone does not substantiate abundance differences; an orthogonal quantitative method (e.g., Western blot, PRM/SRM targeted proteomics) is required or a detailed technical explanation for its

infeasibility must be provided along with an alternative quantitative approach. Additionally, the manuscript should further moderate causal interpretations and explicitly acknowledge unmeasured confounders. With these issues resolved and data availability brought

into compliance, the manuscript may become suitable for publication.

**Author response:** We appreciate the reviewer’s constructive feedback regarding the need for robust quantification. To address this, we have now incorporated RT-qPCR as an independent, orthogonal quantitative approach to validate our proteomic findings. We updated **the Methods section** to include the RT-qPCR analysis as following: “Validation analysis was performed via RT-qPCR on sperm samples from 14 NOR and 16 TER men who showed detectable activity in the TGF-β signalling pathway. The FavorPrep™ Tissue Total RNA Mini Kit (Favorgen Biotech Corp, Taiwan) was used to extract total RNA from the sperm samples following the manufacturer's protocol. The mRNA was reverse transcribed into cDNA using the Maxime™ RT PreMix (Oligo dT Primer) (iNtRON Biotechnology, Korea). The primers for the target genes, including the LTBP1 gene (forward primer 5'-TGAATGCCAGCACCGTCATCTC-3'; reverse primer 5'-CTGGCAAACACTCTTGTCCTCC-3') and the TGFBR1 gene (forward primer 5'-TCAGCTCTGGTTGGTGTCAG-3'; reverse primer 5'-ATGTGAAGATGGGCAAGACC-3'), were used. The glyceraldehyde-3-phosphate dehydrogenase (GAPDH) gene (forward primer 5'-CTCAACGACCACTTTGTCAAGCTCA-3'; reverse primer 5'-GGTCTTACTCCTTGGAGGCCATGTG-3') was used as an internal control to normalise the mRNA expression of the target genes. Each PCR product was amplified from 4 ng of cDNA template using qPCRBIO SyGreen Mix (PCR Biosystems, UK). Quantitative PCR was conducted on a QuantStudio™ 5 Real-Time PCR System (Thermo Fisher Scientific, USA). We used the 2-∆∆Cq method to measure the relative mRNA expression levels of LTBP1 and TGFBR1 genes.” [Lines 142-157].

Our new results have been added to demonstrate a significant increase in mRNA levels for key targets, including *LTBP1* and *TGFBR1* genes, which directly corroborate the trends observed on our proteomic screen and immunocytochemistry. This dual-level validation (mRNA and protein) provides a more comprehensive and quantitative basis for our findings. The manuscript has been updated in **the Results section** as follows: “Validation of LTBP1 and TGFBR1 mRNA expression in human sperm

To validate the results of the proteomic analysis, we quantified the mRNA expression of *LTBP1* and *TGFBR1* genes in the sperm samples. The RT-qPCR results confirmed a significant differential expression of both genes. The relative mRNA expression of these genes was shown in Fig 5. The expression of the *LTBP1* gene in the TER group was significantly increased compared to the NOR group (1.23 ± 0.05 vs. 1.03 ± 0.06, p < 0.01), which is consistent with the proteomic analysis results. Moreover, the expression of the *TGFBR1* gene in the TER group was also significantly increased compared to the NOR group (1.16 ± 0.02 vs. 1.01 ± 0.03, p < 0.001).” [Lines 229-236]. Figure 5 and its caption have been added as follows: “Fig 5. The mRNA expression of LTBP1 and TGFBR1 genes in human sperm. Values are shown as mean ± SEM, TER (n = 16) vs. NOR (n = 14). **p < 0.01 and ***p < 0.001; unpaired Student’s t test.” [Lines 238-240].

The manuscript has been updated in **the Discussion section** as follows: “In this study, the significant upregulation of TGF-βR1 at both the mRNA and protein levels in TER men points toward TGF-β-mediated immune activation, potentially reflecting a response to the higher prevalence of abnormal sperm morphology.” [Lines 332-334] and “We also observed a significant upregulation in LTBP1 expression at both mRNA and protein levels in the sperm of men with TER, which inversely correlated with the percentage of normal sperm morphology.” [Lines 356-358]. **The Conclusion section** has been updated, as stated: “Validation of *LTBP1* and *TGFBR1* genes, key components of the TGF-β signalling pathway, revealed a significant increase in their mRNA expression levels in the sperm of TER men.” [Lines 381-383]. **The Abstract section** has been updated, as stated: “Validation analysis revealed that the mRNA expression levels of *LTBP1* and *TGFBR1* genes were significantly upregulated in the TER group relative to the NOR group.” [Lines 34-36].

In accordance with the Reviewer's suggestion, we have updated **the Discussion section** (Lines 358-362) to explicitly acknowledge potential unmeasured confounders as follow: “While the association between upregulation of TGF-βR1 and LTBP1 and abnormal sperm morphology is compelling; it is important to explicitly acknowledge that our study did not account for certain unmeasured confounders, such as subclinical localized inflammation and individual lifestyle factors. These factors could potentially influence TGF-β signalling and sperm quality.”.

**Response to Editor:**

1. It is advised to deposit all the available data, conduct thorough statistical analyses and to substantiate the differential expression by standard techniques like Western blotting.

**Author response:** We thank the Editor for these constructive suggestions, which have significantly strengthened the manuscript. We have addressed each point as follows:

- **Data deposition:** In the interest of transparency and reproducibility, we have deposited the LC-MS/MS datasets and search result files into the ProteomeXchange repository under the accession IDs JPST004285 and PXD072557 (via jPOST: https://repository.jpostdb.org/preview/7054576026955c0503b3d8, Access key: 9498).
- **Thorough statistical analysis:** The manuscript now explicitly acknowledges that LTBP1 did not survive global FDR correction. However, we provide evidence that its differential expression is robust under alternative statistical frameworks. We justified prioritizing LTBP1 based on its consistent fold-change and nominal p-value, which were independently confirmed through orthogonal RT-qPCR validation.
- **Substantiation of differential expression:** To validate the proteomic findings using an orthogonal quantitative method, we performed RT-qPCR to measure the mRNA expression levels of *LTBP1* and *TGFBR1* genes. While Western blotting was suggested, we decided on RT-qPCR as it provides high sensitivity and a broader dynamic range for quantifying gene expression changes. Our targeted analysis confirmed a significant increase in *LTBP1* and *TGFBR1* mRNA levels, successfully validating the biological relevance of this candidate at the transcriptional level. This cross-validation between the proteome and transcriptome provides strong evidence that the observed changes represent a true biological signal.
