# Supplementary material for: Differential protein expression profiles in human sperm from teratozoospermic and normozoospermic men identify LTBP1 and TGF-βR1 as potential biomarkers within the TGF-β signalling pathway
Source: PLoS One. 2026 Feb 13;21(2):e0342465. doi: 10.1371/journal.pone.0342465 (PMC12904376; doi:10.1371/journal.pone.0342465)
Supplement: Attachment — Submitted filename: PLOS- Feedback .docx [file pone.0342465.s005.docx]

# Reviewer Evaluation of Author Rebuttal

Overall assessment

The authors have substantively and satisfactorily addressed the major concerns raised during peer review. The rebuttal demonstrates clear engagement with reviewer feedback, appropriate corrective actions, and improved methodological transparency. From a reviewer’s perspective, the manuscript is now acceptable for publication, with only minor optional refinements suggested.

1. Data availability and PLOS ONE compliance

The authors have fully addressed data availability requirements. Raw LC-MS/MS data have been deposited in ProteomeXchange with valid accession numbers (JPST004285, PXD072557 via jPOST). Access information is clearly provided, and availability of clinical and MALDI-TOF MS data is transparently stated. This satisfies PLOS ONE’s mandatory data-sharing policies.

2. Proteomics statistics and multiple-testing correction

The response appropriately applies Benjamini–Hochberg false discovery rate correction and transparently reports both nominal p-values and FDR-adjusted q-values. The authors correctly acknowledge that LTBP1 does not survive global FDR correction and have reframed it as a biologically relevant candidate rather than a definitive discovery. Interpretation is cautious and statistically sound.

3. Validation strategy and quantitative rigor

The addition of RT-qPCR as an orthogonal quantitative validation substantially strengthens the manuscript. Methods are clearly described, sample sizes are appropriate, and results demonstrate robust differential expression of both LTBP1 and TGFBR1. While RT-qPCR does not directly quantify protein abundance, the convergent evidence from proteomics, immunocytochemistry, and transcript-level validation is acceptable for a discovery-focused PLOS ONE study.

4. Causal interpretation and confounders

The authors have appropriately moderated causal language and explicitly acknowledged unmeasured confounders, including potential inflammatory and lifestyle factors. Interpretations are now framed as associative rather than causal, aligning with observational study standards.

5. Editorial quality

The rebuttal is clear, professional, and well-structured. Revisions are consistently reflected across the manuscript sections, with precise line references provided.

Minor optional suggestions

These are not required for acceptance:
- Consider explicitly referring to LTBP1 as a “prioritized candidate protein” in the Results.
- Include a brief statement noting that transcript abundance may not always directly reflect protein abundance.

Final recommendation

Accept for publication. All major scientific, statistical, and compliance issues have been satisfactorily addressed, and the manuscript now meets the standards for publication in PLOS ONE.
